# Tetanus—Treated by Hydrate of Chloral—Recovery

**Published:** 1871

**Authors:** W. M. McGalliard

**Affiliations:** Donaldsonville, Louisiana


ARTICLE III.— *Tetanus — Treated by Hydrate of Chloral — Recovery*. By W. M. McGALLIARD, M.D., Donaldsonville, Louisiana.

---

Joseph Perilloux, aged thirteen years, a well-developed farmer's boy, native of the Parish of Assumption, Louisiana, ran a nail into the plantar surface of the left foot, on the 9th of March. The nail was removed, and little inconvenience felt till the 20th, when after a little pain in the foot and limb, well marked symptoms of tetanus were developed on the 25th, sixteen days after the wound. I saw him on the morning of the 25th at 10 o'clock. The characteristic risus sardonicus was well marked ; jaw stiff, but could be opened with the finger so as to allow him to take fluids. He could not articulate ; abdomen hard; appetite good ; bowels constipated ; pulse excited ; temperature 99 ; wound unhealthy ; secreting a small quantity of sanious fluid. Ordered poultice and turpentine to the wound; an injection of oil, turpentine and water; and ten grains of hydrate chloral every two hours till relief is obtained.

27*th*, 10 A. M.—Much better; slept well after taking the first dose of chloral; spasm returned again in the evening; relieved by chloral; slept well during the night; spasm returned again this morning at 6 A. M.; relieved as before. There is now very little stiffness of jaw; abdominal muscles relaxed; can take and swallow fluids with mush with very little difficulty; bowels opened; treatment continued, and chloral to be increased to twenty grains if necessary.

28*th*.—About the same as yesterday, rested well during the night; has had but two spasms since I saw him yesterday. Treatment continued.

29*th*.—Same as yesterday, with pain in back; ordered a blister one by six inches to the spine; other treatment continued, except chloral, which, on account of being so unpleasant, was substituted by a fourth of a grain of morphine every four hours.

30*th*, 12 M.—Much worse; blister produced strangury; has not passed urine for twenty hours; boy much excited, and this condition increased by the anxiety of family and friends. Pulse 90; free perspiration; jaw stiffer; opisthotonos well marked; ordered warm bath and a fourth of a grain of morphine every four hours, and if necessary, to give him eighty grains of chloral, by enema, as he cannot take it by the mouth.

31*st*, 10 A. M.—Better; passed urine after bath; had severe spasm at 11 P. M., when he took, by enema, eighty grains of chloral, after which he slept till morning; jaws easily opened; opisthotonos relieved; stomach softer; pulse 80; skin natural; wound healed; only a drop or two of pus has been noticed since the 26th. Takes chloral well in 20-grain doses by the mouth. Appetite good; bowels opened without enema; treatment continued.

*April* 1*st*.—Patient passed a good night; no severe spasm; general symptoms better; treatment continued.

*April* 2*d*.—Doing well; chloral produces slight intoxication for a short time, after which sound sleep ensues. Appetite good; bowels acted; skin natural; temperature 98½. Ordered chloral to be suspended during the day, and to take a fourth of a grain of morphine if an opiate is necessary.

*April* 3*d*.—Not so well; ate very heartily yesterday, which produced colic, aggravating his symptoms very much. Took fourth of a grain of morphine per enema at midnight, after which he slept well. Has pain in abdomen; no appetite; pulse 90; temperature 100; skin moist. Ordered ten grains chloral, with half an ounce of castor oil by stomach, to be followed by large enema of water in two hours, and chloral to be repeated when necessary in ten or twenty-grain doses.

*April* 4*th*.—Better; bowels acted once last night, and copiously this morning; pulse 84; skin natural; spirits good; appetite returned. Took oil and chloral, but owing to his distaste to chloral, did not repeat it till midnight, after which he slept well. Ordered chloral to be continued with moderate amount of nourishment.

*April 8th.*—Owing to indisposition on my part, I did not see the patient since the 4th, since which time he has improved slowly. There is some stiffness of jaw and body. He eats and sleeps well; takes chloral when necessary to quiet him; and when awake amuses himself by playing upon his violin, of which he has been quite fond since his disease moderated. Since the above date I have not seen him, but was informed by his mother about ten days after, that he had completely recovered. In this case, chloral seemed to act as a sedative and as a cathartic by relaxation without in the slightest degree affecting the appetite or deranging digestion. The patient occasionally manifested an intolerance of the chloral, but by suspending it for a while and giving it in smaller doses, and in the meantime controlling the severe symptoms by some other sedative, the same happy effect was obtained as in the beginning of its use.—*Richmond and Louisville Medical Journal.*

